# Mapping of CELF1–RNA interactions reveals post-transcriptional control of lens development

**DOI:** 10.1093/narmme/ugag037

**Published:** 2026-08-05

**Authors:** Justine Viet, Matthieu Duot, Agnès Méreau, Yann Audic, Iwan Jan, David Reboutier, Catherine Le Goff-Gaillard, Sarah Y Coomson, Salil A Lachke, Carole Gautier-Courteille, Luc Paillard

**Affiliations:** Univ Rennes, CNRS, IGDR (Institut de Génétique et Développement de Rennes), UMR 6290, F-35000 Rennes, France; Univ Rennes, CNRS, IGDR (Institut de Génétique et Développement de Rennes), UMR 6290, F-35000 Rennes, France; Department of Biological Sciences, University of Delaware, Newark, DE 19716, United States; Univ Rennes, CNRS, IGDR (Institut de Génétique et Développement de Rennes), UMR 6290, F-35000 Rennes, France; Univ Rennes, CNRS, IGDR (Institut de Génétique et Développement de Rennes), UMR 6290, F-35000 Rennes, France; Univ Rennes, CNRS, IGDR (Institut de Génétique et Développement de Rennes), UMR 6290, F-35000 Rennes, France; Univ Rennes, CNRS, IGDR (Institut de Génétique et Développement de Rennes), UMR 6290, F-35000 Rennes, France; Univ Rennes, CNRS, IGDR (Institut de Génétique et Développement de Rennes), UMR 6290, F-35000 Rennes, France; Department of Biological Sciences, University of Delaware, Newark, DE 19716, United States; Department of Biological Sciences, University of Delaware, Newark, DE 19716, United States; Univ Rennes, CNRS, IGDR (Institut de Génétique et Développement de Rennes), UMR 6290, F-35000 Rennes, France; Univ Rennes, CNRS, IGDR (Institut de Génétique et Développement de Rennes), UMR 6290, F-35000 Rennes, France

## Abstract

Precise post-transcriptional regulation of gene expression is essential for vertebrate lens development. Disruption of the gene encoding the RNA-binding protein CELF1 leads to early-onset cataract in mice. Here, using iCLIP-seq in lenses, we mapped transcriptome-wide CELF1-binding sites, revealing interactions with the 3′UTRs of key transcripts involved in lens development and pathology like Gja8, *Jag1*, Maf, Pax6, or Prox1. Integrated analysis with transcriptomic data and luciferase reporter assays demonstrated that binding of CELF1 protein represses its target mRNAs by destabilizing transcripts and/or inhibiting their translation. Indeed, the cataract-linked genes *Maf* and *Gja8* are upregulated in *Celf1*-cKO lenses. In *Xenopus laevis*, overexpression of maf resulted in abnormal lens structure and eye morphology, confirming the developmental relevance of CELF1-mediated repression. Our findings uncover a post-transcriptional network in which CELF1 controls lens morphogenesis by limiting the expression of critical genes at the mRNA level to achieve their proper dosage.

## Graphical Abstract

**Graphical Abstract:**
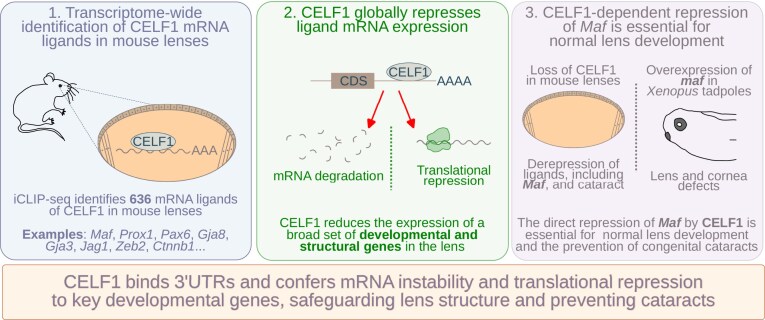

## Introduction

The ocular lens is a biconvex organ located behind the iris and in front of the retina. Along with the cornea, the lens focuses light onto the retina, a function that depends critically on its transparency and precisely controlled refractive index. The lens is composed mainly of highly differentiated, non-proliferative lens fiber cells arranged concentrically. Fibers are surrounded anteriorly by a monolayer of epithelial cells. These epithelial cells divide in the proliferation zone and, when they reach the equatorial region of the lens, they initiate differentiation into fiber cells. The specific optical properties of the lens are achieved through multiple mechanisms, including the elimination of organelles and nuclei in fiber cells and the accumulation of highly refractive proteins known as crystallins [1–4].

Loss of lens transparency is defined as a cataract, a major age-related eye disease and a leading cause of blindness worldwide [5–7]. Cataracts result from the formation of light-scattering protein aggregates in the lens. They essentially affect older individuals as such aggregates gradually accumulate with age. Environmental conditions such as UV light and oxidative stress promote this process [8]. However, cataracts can also be congenital, typically with a genetic basis [7, 9]. The Cat-Map database catalogs cataract-associated genes and loci in vertebrates [10, 11], listing over 500 genes as of its February 2025 release. These include genes that encode crystallin proteins, membrane proteins such as connexins and the aquaporin MIP, or transcription factors essential for lens development such as *PAX6, MAF, FOXE3*, and *HSF4* [11].

Beyond transcriptional regulators, post-transcriptional mechanisms play a pivotal role in mediating spatiotemporal control of gene expression during lens development. RNA-binding proteins (RBPs), in particular, have emerged as critical regulators [12, 13]. For example, *TDRD7* was first implicated in cataract through human balanced chromosomal rearrangements and inherited mutations, and validated in animal models [14–16]. A large genome-wide association study linked several RBP genes, including *THOC7, QKI, CAPRIN2*, and *RBFOX1*, to cataract [17]. Functional studies have shown that conditional inactivation of *Caprin2* or *Qki* in the mouse lens leads to cataract, the latter due in part to disrupted cholesterol biosynthesis [18–20]. Interestingly, although QKI is typically characterized as an RBP [21], it may function at the transcriptional level in the lens [20]. Recent transcriptome/proteome analyses have expanded the list of RBPs expressed in the lens [22].

CELF1 (CUGBP, Elav-like family member 1) is a well-known RBP recognized for its role in alternative splicing regulation [23–27]. It also promotes transcript destabilization via binding to the 3′ untranslated regions (3′UTRs), targeting genes such as *JUN, JUNB*, or *TNFRS1B* [28–31]. It also controls mRNA translation of mouse *Cyp19a1* as well as of several human genes involved in epithelial-to-mesenchymal transition [32, 33]. Conditional deletion of *Celf1* gene in the mouse lens causes early-onset cataracts [34]. In human, CELF1 is indirectly implicated in cataract through Myotonic Dystrophy, type 1 (DM1), a multisystem disorder that causes cataracts among other symptoms. The genetic basis of DM1 is a CTG repeat expansion in the *DMPK* gene, which leads to increased CELF1 activity and reduced MBNL1 function by still incompletely understood mechanisms. As CELF1 and MBLN1 are both RBPs involved in splicing control, this would disrupt splicing regulation and cause the symptoms of DM1 [23–25]. A related disease, Myotonic Dystrophy, type 2 (DM2), also presents with cataracts and is caused by repeat expansion in the *CNBP* gene, which encodes a nucleic acid (including RNA) binding protein [35, 36].

In the lens, a candidate gene approach revealed that CELF1 regulates the two key transcription factors PAX6 and PROX1 at a post-transcriptional level [37]. A recent transcriptomic study further showed that *Celf1* inactivation disrupts lens differentiation, as revealed by the downregulation of multiple lens terminal differentiation genes [38]. However, the direct molecular mechanisms by which the lack of CELF1 results in cataracts remain unclear. Specifically, the full repertoire of RNAs bound by CELF1 in the lens and the functional consequences of these protein–RNA interactions are unknown.

To address this gap, we performed transcriptome-wide identification of CELF1-bound RNAs in mouse lenses using iCLIP-seq, a method that maps RNA–protein interactions at nucleotide resolution [39, 40]. By performing an integrated analysis of iCLIP-seq data with gene expression profiling data on *Celf1*-cKO lenses, and mechanistically testing key candidates with luciferase reporter assays, we show that CELF1 functions as a global post-transcriptional repressor in the lens. Using a stepwise prioritization pipeline, we identify high-confidence CELF1 ligands whose increased expression in the absence of CELF1 likely contributes to cataract. Among them, we validate *Maf* and *Gja8* as key CELF1 ligands, showing that they are upregulated in the absence of CELF1 *in vivo*. Overexpression of *Maf* in *Xenopus* larvae elicites lens defects reminiscent of those observed in *Celf1*-deficient mice, indicating that tight regulation of *Maf* by CELF1 is important for normal lens development. Together, these findings (i) reveal for the first time the *in vivo* ligands of CELF1 in a specific organ, (ii) demonstrate CELF1’s global repressive role in lens gene expression, and (iii) identify candidate genes whose increased expression is likely pathogenic, offering a complementary resource to traditional loss-of-function screens for cataract gene discovery.

## Materials and methods

### Ethics statement

Mouse experiments were conducted either at the University of Rennes (France) or the University of Delaware (USA). In Rennes, mice were bred in the Biosit animal facility (Arche) approved by the French animal care agency (Direction des Services Vétérinaires, approval number A3523840). All procedures were performed following standard guidelines. At the University of Delaware, mice were bred and maintained in the Center for Animal Research facility. All animal protocols were reviewed and approved by the Institutional Animal Care and Use Committee (IACUC, protocol number 1226). All procedures conformed to the ARVO Statement for the Use of Animals in Ophthalmic and Vision Research.

*Xenopus laevis* experiments were conducted at the University of Rennes. Animals were housed in the IGDR facilities, accredited by the French animal care agency (Direction des Services Vétérinaires). Procedures were approved by the local ethics committee and authorized by the Ministry of Research (APAFIS #45521, 2023).

### iCLIP-seq of CELF1

Lenses were dissected from euthanized 2-month-old mice, placed on Parafilm on ice, UV-irradiated (254 nm, 3 × 4000 µJ/cm²), ground in 100 µl of PXL buffer per lens (50 mM Tris–HCl pH 7.4, 0.1 M NaCl, 1% NP40, 0.1% SDS, 0.5% DOC, plus DTT, RNasin, and protease inhibitors, Sigma P8340) and stored at −80°C. Dissections and irradiations were performed on 25 mice per iCLIP-seq experiment, one mouse at a time to preserve lens freshness. The subsequent steps were essentially as described [39], except that the L3 primer was directly radiolabeled and adenylated to give L3-App via (i) phosphorylation with [γ-32P] ATP and PNK (New England Biolabs), and (ii) adenylation of radiolabeled L3 with ATP and Mth RNA ligase (New England Biolabs). Briefly, lenses lysates were treated with Turbo DNase and RNase T1 (CLIP1: 2.4 U RNase T1/mg protein extract, Sigma; CLIP2: equivalent of 100 U RNase T1/mg protein extract, Ambion) for 10 min at 37°C. CELF1–RNA complexes were immunoprecipitated at 4°C with the 3B1 monoclonal antibody (Santa Cruz sc-20003), dephosphorylated, and ligated to radiolabeled, pre-adenylated L3-App. The 5′ end labeling step was omitted. Next, RNA–protein complexes were resolved on NuPAGE Bis–Tris gels, transfered to pure nitrocellulose membranes, and RNA was recovered from the membrane by proteinase K digestion. Reverse transcription, circularization-linearization, and polymerase chain reaction (PCR) amplification were performed as described [39]. Libraries were sequenced on an Illumina platform (strand-specific, single-end, 50 bp reads).

### Analysis of **iCLIP-seq data and statistical analyses**

Raw reads were processed using iCount 2.0.1 (https://icount.dev/). Reads were trimmed to remove adaptors, demultiplexed and filtered to retain those ≥19 nucleotides. Mapping was performed against the mouse genome (GRCm38) using STAR [86], keeping only uniquely mapped reads. Crosslink sites were defined as the terminal positions of the reads and scored by read count. Significance was calculated as in FOX2 studies [42], using a false discovery rate (FDR) calculated over ± 3 nt windows compared to random background. Only sites with FDR < 0.05 were retained and termed peaks. Peaks < 20 nt apart were clustered, and cluster scores were calculated as the sum of peak and adjacent signal scores. The data have been deposited in NCBI’s Gene Expression Omnibus and are accessible as GSE313211.

iCLIP-seq data, as well as other quantitative data, were analyzed with R. Plots were prepared with the ggplot2 package. A fully annotated script is available at https://zenodo.org/records/20830191.

### CompBio-based analysis of iCLIP-seq targets

A computational tool CompBio (developed at Washington University https://gtac-compbio-ex.wustl.edu/) was used to apply language processing and a curated biological dictionary to extract the concepts enriched in the candidates identified by the iCLIP-seq assays. CompBio performs *de novo* analysis of a given dataset using publicly available scientific literature (currently covering > 30 million PubMed abstracts and > 3 million full-text articles). CompBio reveals the biological “concepts” that are enriched in the dataset. As previously described, a normalized enrichment score (NEScore) indicates the significance of the identified themes and statistical confidence in the enrichment of a theme relative to random gene sets is indicated by *P* values. CompBio was used to analyze 636 genes [227 commonly found in both CLIP1 and CLIP2 plus those exclusively found in CLIP1 (380) and CLIP2 (29)]. Themes that had NEScore ≥ 1.3 and *P* < 0.1 were considered significant. An automated theme annotation feature in CompBio was used (and modified where necessary) to underscore key biological processes.

### Luciferase and RT-qPCR assays

3′UTRs were PCR-amplified from reverse-transcribed mRNA of mouse lenses using gene-specific primers (Supplementary Table S2). PCR products were cloned downstream of the firefly luciferase gene in the *pmirGlo* vector (Promega), using Gibson Assembly (NEBE2611S/L). Mutant constructs lacking CELF1-binding clusters were generated by amplifying 5′ and 3′ fragments separately (primer sequences in Supplementary Table S2). Both fragments were inserted simultaneously into *pmirGlo*. Transfections and luciferase assays followed previously described methods [32].

For RT-qPCR, nonclonal populations of stably transfected cells were generated by adding 800 µg/ml Geneticin 72 h after transfection. After two weeks of selection, the Geneticin concentration was reduced to 400 µg/ml, as previously described [32]. Cells were cultured in six-well plates, and total RNA was extracted using the Nucleospin RNA kit (Macherey-Nagel), which includes on-column DNase according to the manufacturer’s instructions. RNA concentration and purity were assessed by NanoDrop spectrophotometry.

For reverse transcriptions, 500 ng of total RNA were mixed with 1 µl of 10 mM dNTPs and 1 µl of random primers (3 µg/µl; Invitrogen 48190011) in a final volume of 12 µl. The mixture was heat-denatured (65°C, 5 min) and immediately chilled on ice. We added 1 µl of RNaseOUT (Invitrogen 10777019), 2 µl of 0.1M DTT, 4 µl of 5× SuperScript buffer, and 0.5 µl of SuperScript II Reverse Transcriptase (Life Tech 18064). Reverse transcription was at 42°C for 90 min, followed by enzyme inactivation at 72°C for 10 min.

Quantitative PCR was performed in 10 µl reactions containing 5 µl Power Sybr Green PCR Master Mix (Applied Biosystems, 4367659), 0.3 µl of each primer (10 µM; primer sequences are in Supplementary Table S2), 2.4 µl of water, and 2 µl of 20-fold diluted cDNA. PCR conditions were 95°C for 2 min, then 40 cycles of 95°C for 15 s and 60°C for 1 min. RT-minus controls were included systematically, and experiments were retained only when the difference in Ct between RT+ and RT− samples exceeded 5 cycles, indicating negligible plasmid contamination. Firefly luciferase mRNA levels were normalized to Renilla luciferase mRNA levels. Each biological replicate (independent cell culture) corresponds to the median of at least 4 technical qPCR replicates performed from the same reverse transcription.

### Immunofluorescence on mouse eye

Eyes from postnatal day 15 (P15) were fixed in 4% paraformaldehyde (PFA) for 30 min on ice, followed by incubation in 30% sucrose overnight at 4°C, embedded in OCT (Tissue-Tech), and sectioned at 14 μm. Sections were blocked in 5% chicken serum (Abcam, Cambridge, UK) with 0.3% Triton for 1 h at room temperature. The sections were incubated overnight at 4°C with the following primary antibodies: CELF1 (Santa Cruz Sc-20003, 1:500), MAF (Abclonal A12720, 1:2500), and GJA8 (Life Technologies PA5-11644, 1:1000). Sections were washed and incubated with Alexa Fluor 594-conjugated secondary antibody (1:1000; Life Technologies, Carlsbad, CA) and 4′,6-diamidino-2-phenylindole (DAPI, 1:1000) for 1 h at room temperature. Slides were mounted using mounting media (ProLong Gold antifade reagent, Invitrogen P36930) and imaged on a Zeiss LSM 880 confocal microscope configured with Diode/Argon laser (405 nm, 488 nm and 594 excitation lines). Image brightness/contrast was adjusted identically across all conditions using Adobe Photoshop. Signal quantification was performed using Fiji (ImageJ). The figures show representative control and cKO littermates. Similar results were obtained in three independent control/cKO littermate pairs analysed for each condition.

### Cell culture and immunocytofluorescence

21EM15 mouse lens epithelial cells (from Dr John Reddan, Oakland University, Rochester, MI, USA) were cultured in DMEM with 4.5 g/l glucose, L-glutamine, and sodium pyruvate (Life Technologies, Carlsbad, CA, USA) with 10% FBS (Eurobio, Les Ulis, France) and 1% penicillin–streptomycin (Life Technologies, Carlsbad, CA, USA) at 37°C, 5% CO_2_. Cells were split three times weekly and typically reached 80% confluence within three days. The stable *Celf1*-knockdown 21EM15 derivative, generated via lentiviral transduction with *Celf1*-specific shRNA, has been described [34].

For immunostaining, 5 × 10^4^ cells/cm^2^ were seeded on glass coverslips. After 24 h, cells were fixed in 4% PFA in MES buffer (10 mM MES buffer, 138 mM KCl, 3 mM MgCl_2_, 0.1 g/ml sucrose, and 2 mM EGTA, pH 6.1) for 10 min. They were permeabilized with 0.1% Triton X-100 and blocked in 1% BSA in PBST for 1 h at room temperature. Primary antibodies in PBS–BSA include Rabbit anti-MAF (1:500; Abcam 77071), rabbit anti-PAX6 (1:200; Abcam Ab2237), and rabbit anti-PROX1 (1:200; Biolegend, 925201). The cells were incubated with antibodies overnight at 4°C. After washes, cells were incubated with the secondary antibodies (Alexa Fluor 488 or 555 anti-rabbit; 1:1000, Invitrogen) for 1 h at RT. Coverslips were mounted with ProLong Gold (Invitrogen) containing 1 µg/ml DAPI (Sigma–Aldrich). Imaging was done using a Nikon NIE microscope with a ×40 objective.

### *Xenopus* embryo manipulations and histological methods

*X. laevis maf.L* ORF was PCR-amplified from reverse-transcribed *Xenopus* embryo mRNA with the primers given in Supplementary Table S2. The PCR product was cloned into BglII-NotI cleaved pT7TS vector [31], using Gibson Assembly (NEBE2611S/L). The plasmid was linearized with BamH1 before *in vitro* transcription (Message Machine T7, Life Technologies AM1344). One dorsal blastomere of four-cell *X. laevis* embryos was injected with 1.25 ng Alexa 568-conjugated dextran (Life Technologies D22912) and 1.5 fmol *maf* mRNA. Embryos were sorted according to injection side after 2 days at 22°C, and analyzed at day 3 for eye malformation.

For histology, embryos at stages 39/40 were fixed in Carnoy for 2 h at room temperature. They were dehydrated (ethanol 100% Eosin 20 min, ethanol 100% 20 min twice, and butanol 30 min twice) and paraffin-embedded as described [59]. Horizontal sections (7 µm) were stained with hematoxylin and eosin.

## Results

### iCLIP-seq identifies mRNA ligands of CELF1 in mouse lenses

To identify the RNA ligands of CELF1 at the transcriptome-wide level, we performed iCLIP-seq experiments on mouse lenses as described [39, 40]. Briefly, lenses freshly dissected from 2-month-old mice were UV-irradiated to induce covalent RNA–protein crosslink, lysed, gently treated with RNase, and subjected to immunoprecipitation with an anti-CELF1 antibody. RNA co-immunoprecipitated with CELF1 was labeled by ligation of a radioactive oligonucleotide. RNA–protein complexes were separated by electrophoresis, transferred to a pure nitrocellulose membrane, and visualized by autoradiography.

To validate the specificity of the procedure, we performed a series of controls (Supplementary Fig. S1A–C). A radiolabeled complex migrating at approximately 60 kDa was detected only in immunoprecipitations from wild-type (*Celf1*^+/+^) or heterozygous (*Celf1*^+/-^) lenses (Supplementary Fig. S1A, lanes 4–5; quantifications in Supplementary Fig. S1B). No signal was detected in immunoprecipitates from *Celf1*-knockout lenses [41] (lane 6), in the absence of CELF1 antibody (lane 1), without UV crosslinking (lane 2), or without RNase treatment (lane 3). RNA recovered by proteinase K treatment from the excised ∼60 kDa complex yielded fragments of approximately 100–150 nucleotides only under conditions where the CELF1–RNA complex was present (Supplementary Fig. S1C). These controls confirm that the recovered RNA specifically originates from CELF1-associated transcripts.

The RNAs recovered from the membrane were reverse-transcribed using the ligated oligonucleotide, amplified and deep sequenced. We then repeated the experiment using a higher RNase concentration to increase stringency. As expected, this second experiment (CLIP2) yielded fewer total and uniquely mapped reads than the first experiment (CLIP1) (Fig. 1A).

**Figure 1.**
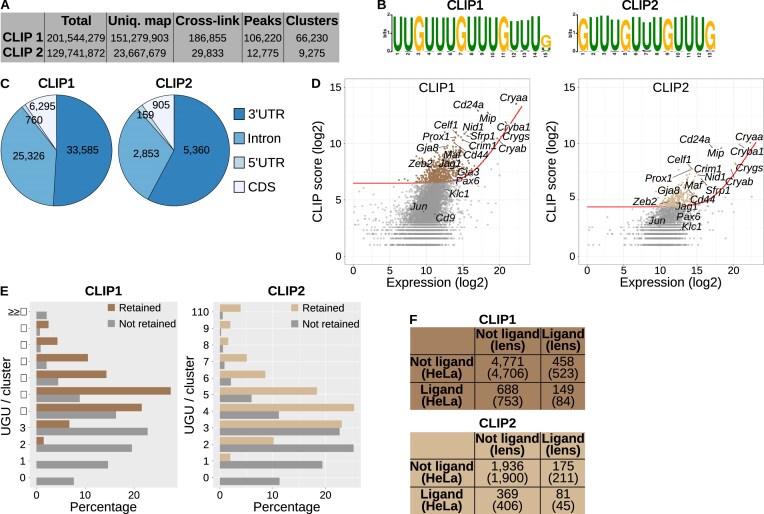
iCLIP-seq identifies CELF1-binding sites in mouse lenses. (**A**) Summary of iCLIP-seq data showing the number of total reads, uniquely mapped reads, crosslink sites, significant crosslink sites (peaks) and peak clusters, as defined in the text, for the two iCLIP-seq experiments. (**B**) Most significantly enriched sequence motifs identified within CLIP1 and CLIP2 binding clusters (*P* = 2.6e-10 and 4.3e-7, respectively). (**C**) Distribution of CELF1 binding clusters across gene regions. Left CLIP1 (33 585 clusters in the 3′UTRs of 6066 genes); right CLIP2 (5360 clusters in the 3′UTRs of 2561 genes). (**D**) CLIP scores in 3′UTRs plotted against gene expression levels (log scales). Each dot represents one gene. Genes retained as CELF1 ligands based on their high CLIP scores relative to expression levels (red lines) are shown in dark brown (CLIP1, *N* = 607) and light brown (CLIP2, *N* = 256). (**E**) Distribution of UGU trinucleotide numbers within CELF1 binding cluster located in 3′UTRs. For genes with multiple clusters, only the cluster with the highest UGU count was retained. (**F**) Contingency tables comparing CELF1 ligands identified in mouse lenses and HeLa cells. CLIP1: 6066 genes with at least one CELF1 binding cluster in the 3′UTR, of which 607 were retained as ligands. CLIP2: 2561 genes with at least one cluster in the 3′UTR, of which 256 were retained as ligands. Expected counts under independence are indicated in parentheses. *P* = 9.6e-16 (CLIP1) and *P* = 7.8e-10 (CLIP2), chi-square test.

In iCLIP-seq, reads terminate at the site where reverse transcriptase is halted by the crosslinked protein, enabling identification of binding sites at near-nucleotide resolution [40]. We identified individual cross-link sites, whose scores correspond to the number of reads terminating at each position. Statistically significant crosslink sites (“peaks”) were detected by comparison with randomized distributions [42], and neighboring peaks were grouped into clusters. This analysis revealed over 66 000 and 9000 CELF1 binding clusters in CLIP1 and CLIP2, respectively (Fig. 1A).

Because the two experiments differed substantially in sequencing depth and cluster number, direct cluster-to-cluster comparisons were not appropriate. Instead, we evaluated reproducibility by determining the extent to which CLIP2 clusters overlapped CLIP1 clusters. We found that 2902 of the 9275 CLIP2 clusters shared at least one nucleotide with a CLIP1 cluster. To assess the significance of this overlap, we performed a gene-centered randomization test in which CLIP2 clusters were shuffled within their parent genes 1000 times before overlap recalculation [43]. The observed overlap greatly exceeded that expected by chance (observed = 2902; mean randomized overlap = 268; *P* < 0.001; Supplementary Fig. S1D), demonstrating strong reproducibility between the two datasets.

As an independent validation, we searched for sequence motifs enriched within CELF1-binding clusters using STREME [44]. The most significantly enriched motifs were highly similar in CLIP1 and CLIP2 (Fig. 1B, *P* = 2.6e-10, CLIP1; *P* = 4.3e-07, CLIP2) and consisted of repeated GU-rich sequences containing multiple UGU trinucleotides, consistent with the known binding preference of CELF1 [45]. Additional enriched motifs showed similar concordance between datasets (Supplementary Fig. S2). Together, the highly significant overlap between CLIP1 and CLIP2 clusters and the recovery of the expected CELF1-binding motif support the robustness of the identified binding sites.

We next examined the genomic distribution of CELF1-binding clusters. We found that >50% of these clusters localize to 3′UTRs in both datasets (Fig. 1C). We therefore focused subsequent analyses on these regions. Significant 3′UTR clusters were detected in 6066 genes in CLIP1 and 2561 genes in CLIP2. Only 199 CLIP2 genes are absent in CLIP1, implying that the higher-stringency dataset was largely included within the lower-stringency dataset. The complete dataset comprising 6265 genes with at least one significant 3′UTR binding cluster is provided in Supplementary Table S1.

Because all 6265 genes exhibited at least one significant CELF1-binding clusters, we next sought to identify the most likely functional ligands. For each gene, we defined a “CLIP score” as the sum of scores of CELF1 binding clusters within its 3′UTR. We observed that CLIP score is significantly correlated with gene expression, based on RNA-seq from wild-type lenses [38] (*R*^2^ = 0.27, CLIP1; *R*^2^ = 0.24, CLIP2), as expected because highly abundant transcripts have a greater probability of interacting with RBPs. To correct for this effect, we selected the 10% of genes with the largest positive residuals from the CLIP score-versus-expression regression. This yielded 607 candidate ligands in CLIP1 and 256 in CLIP2 (Fig. 1D and Supplementary Table S1). Notably, several known actors of lens biology were identified in both datasets, including *Prox1, Gja8, Maf, Jag1, Mip*, the crystallin genes *Cryaa, Cryba1, Crygs* or *Cryab*, or *Celf1* itself. Among previously reported CELF1 ligands [26, 27, 31, 45], *Cd44* was recovered, but *Jun, Klc1*, and *Cd9* were not detected using our thresholds.

As noticed above, CELF1 binding sites are enriched in UGU trinucleotide motifs (Fig. 1B) [45]. To further evaluate the validity of this filtering strategy, we examined UGU enrichment within retained and non-retained ligands. Retained ligands displayed a striking enrichment in UGU-containing binding clusters (Fig. 1E). For example, in CLIP1, 598 of the 607 retained genes have clusters with at least 3 UGUs, whereas only 377 would be expected by chance (*P* = 4.3e-84, chi-square test). Similar enrichment was observed in CLIP2 (*P* = 4.2e-40). Thus, selecting ligands on the basis of CLIP score relative to transcript abundance substantially enriches for high-confidence CELF1 targets.

Finally, we compared our lens-derived CELF1 ligands with those reported in human HeLa cells [46]. Of the 607 CLIP1 ligands and 256 CLIP2 ligands, 149 and 81, respectively, were also reported in HeLa cells, significantly exceeding random expectation (Fig. 1F and Supplementary Table S1). Hence, although the repertoires of CELF1 ligands cannot largely overlap between two highly different cell types that express dissimilar sets of genes, the enrichment of previously identified CELF1 ligands within both CLIP1 and CLIP2 support the biological relevance and reproducibility of our findings.

### CELF1 is a global post-transcriptional repressor in the lens

From CLIP1 and CLIP2, we identified 607 and 256 CELF1-bound mRNAs, respectively, with 227 shared between them (Fig. 2A). Thus, the higher-stringency dataset (CLIP2) is largely contained within CLIP1, with 88.7% (227/256) of CLIP2 ligands also detected in CLIP1. We defined two gene sets. The intersection includes 227 genes, shared between both CLIP1 and CLIP2, representing high-confidence ligands of CELF1. The union includes 636 genes, 227 commonly found in both CLIP1 and CLIP2 plus those exclusively found in CLIP1 (380) and CLIP2 (29) (Fig. 2A and Supplementary Table S1). To further assess the consistency of the two datasets, we compared CLIP scores between experiments. Despite differences in RNase stringency, CLIP1 and CLIP2 scores were significantly correlated (*R*² = 0.38 on log-transformed values; Fig. 2B), supporting the robustness of ligand identification across experimental conditions.

**Figure 2.**
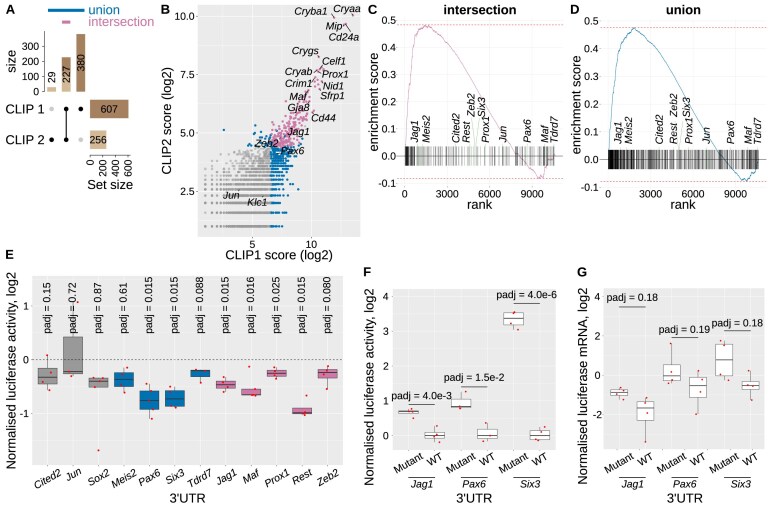
CELF1 represses gene expression through effects on mRNA levels and translation. (**A**) Upset plot showing the overlap between CELF1 mRNA ligands identified from 3′UTR binding in CLIP1 and CLIP2. The intersection (magenta, *N* = 227) includes mRNAs bound in both experiments, and the union (blue, *N* = 636) includes mRNAs bound in at least one. (**B**) Comparison of CLIP1 and CLIP2 scores, log scale. Magenta genes in the intersection; blue genes in the union but not the intersection. (**C** and **D**) GSEA-inspired enrichment analysis. Genes were ranked by signed FDR (−log10 scale) for differential expression between wild-type and *Celf1-*cKO lenses [38]. Most significantly upregulated genes in *Celf1-*cKO lenses are on the left; most significantly downregulated genes on the right. Vertical lines indicate CELF1 ligands belonging to the intersection (C) or the union (D). Selected genes used in subsequent experiments are indicated. (**E**) Luciferase reporter assays using constructs with the indicated 3′UTRs downstream of firefly luciferase. The same plasmid also encodes a renilla luciferase. Luciferase activity was measured following co-transfection by the luciferase plasmid and a plasmid driving the expression of *Celf1* or *H2B* as a control. Luciferase was normalized sequentially: (i) to renilla luciferase (*F*/*R*), (ii) to the *F*/*R* ratio of a control (plasmid) 3′UTR, and (iii) to the *F*/*R* of cells transfected control with H2B. Values significantly below 0 indicate CELF1-dependent repression. *padj*, Benjamini–Hochberg adjusted *P*-value, Student’s *t*-test (null hypothesis: mean = 0). Gray, 3′UTRs from non-ligands; blue, 3′UTRs identified in a single CLIP dataset; magenta, 3′UTRs identified in both CLIP experiments (intersection). (**F**) Luciferase assays comparing wild-type and mutant 3′UTRs. Luciferase was normalized sequentially to renilla luciferase (*F*/*R*) and to the *F*/*R* ratio of a control (plasmid) 3′UTR, in the absence of any overexpression co-transfected plasmid. (**G**) Relative mRNA levels of the reporter transcripts analysed in (F). In (E–G), each dot represents one biological replicate corresponding to the median of at least 4 technical replicates.

We next integrated these datasets with transcriptomic data comparing wild-type and *Celf1*-conditional knock-out (cKO) lenses [38]. We used a GSEA-inspired enrichment analysis [47]. Briefly, all genes were ranked according to their differential expression between wild-type and cKO lenses, using a signed significance score derived from the FDR, with the most significantly upregulated genes positioned at one end of the ranking and the most significantly downregulated genes at the other. We then assessed whether CELF1 ligands occurred preferentially near either end of this ranked list. The enrichment score (ES) corresponds to the maximum deviation of a running-sum statistic from zero as the ranked list is traversed, increasing when a CELF1 ligand is encountered and decreasing otherwise. Using this approach, we observed that the ligands of CELF1 were highly enriched among the genes most significantly upregulated in the absence of CELF1 (Fig. 2C and D). This enrichment was observed for both the intersection (ES = 0.48, *P* = 4.2e-09) and the union (ES = 0.48, *P* = 1.8e-22), strongly suggesting that CELF1 broadly limits the steady-state abundance of its ligand mRNAs in the lens.

We next investigated whether CELF1 affects gene expression beyond this effect on mRNA stability. We performed luciferase reporter assays upon *Celf1* overexpression using 3′UTRs from 12 genes (Fig. 2E). These included 3′UTRs from (i) 3 genes not identified as CELF1 ligands (*Cited2, Jun, Sox2*), (ii) 4 genes identified as CELF1 ligands in a single CLIP dataset (*Meis2, Six3, Tdrd7, Pax6*), and (iii) 5 genes identified as CELF1 ligands in both CLIP experiments (*Jag1, Maf, Prox1, Rest, Zeb2*). In these experiments, negative values indicate repression of reporter expression by *Celf1* overexpression [32]. As expected, none of the non-ligand 3′UTRs were significantly repressed. In contrast, repression was observed for 2 of the four single-dataset ligands (*Six3, Pax6*), and for 4 of the 5 high-confidence ligands: *Jag1, Maf, Prox1, Rest*.

To confirm that repression is due to CELF1 binding, we examined the distribution of CELF1 binding clusters along these 3′UTRs. While CELF1 spreads all along the 3′UTR for most of them, we identified discrete high-density regions of CELF1 clusters in *Jag1, Pax6*, and *Six3* (Supplementary Fig. S3). Deleting these regions from the 3′UTRs significantly increased luciferase activity (Fig. 2F), demonstrating that CELF1 directly represses gene expression through these binding sites.

We next investigated whether CELF1-mediated repression reflected mRNA destabilization, translational inhibition, or both. To address this question, we quantified reporter mRNA levels for wild-type and mutant *Jag1, Pax6*, and *Six3* 3′UTRs (Fig. 2G). Despite the increased luciferase activity observed after deletion of CELF1-binding regions (Fig. 2F), we failed to detect any significant difference in reporter mRNA levels. These results indicate that CELF1 represses these transcripts primarily at the translational level. Additional evidence for translational repression emerged from comparison with the lens transcriptomic data. Several validated CELF1 targets, including *Maf, Pax6, Prox1*, and *Rest*, showed significant repression in luciferase assays but were not among the most strongly upregulated transcripts in *Celf1*-cKO lenses and did not contribute to the GSEA leading edge (Fig. 2C and D). In GSEA, the leading edge corresponds to the subset of genes that appears before the enrichment score reaches its maximum and thus contributes most strongly to the enrichment signal. The absence of these genes from the leading edge indicates that their mRNA levels are not markedly increased following CELF1 deletion. Nevertheless, their expression remained sensitive to CELF1 in luciferase assays, suggesting that CELF1 can repress these targets independently of major changes in mRNA abundance. Consistent with this interpretation, deletion of CELF1-binding regions from the *Jag1, Pax6*, and *Six3* 3′UTRs increased reporter activity without significantly altering reporter mRNA levels. Together, these findings indicate that RNA-seq alone probably underestimates the extent of CELF1-mediated repression and support a dual role for CELF1 in controlling both mRNA stability and translation in the lens.

### CompBio-based analysis of iCLIP-seq-based CELF1 mRNA ligands

We used a computational tool called CompBio (Comprehensive Multi-omics Platform for Biological InterpretatiOn tool), a literature-based enrichment platform that identifies biological themes from large gene sets, to identify biological themes enriched in the CLIP union dataset (636 genes) [48–53]. CompBio identified themes related to “lens development, lens homeostasis, cataract,” “Connexins, lens fiber biology,” and “Basement membrane, extracellular matrix (ECM)” that are related to lens biology, suggesting that CELF1 directly binds mRNAs that are of functional significance in the lens (Fig. 3). Among other themes, the “connexins, lens fiber biology” theme may help explain the cellular vacuole defects observed in *Celf1*-cKO lenses [34]. Likewise, the themes “cytoskeletal remodeling, cell adhesion” and “actin cytoskeletal reorganization, cell–matrix adhesion” are in agreement with the cytoskeletal defects observed in the *Celf1*-cKO lenses [34]. Interestingly, “Post-transcriptional regulation, RBP-based RNA metabolism,” or “cytoskeleton, RBP-based control, Notch pathway components” were also identified as themes, supporting a broad role for CELF1 in coordinating RNA regulatory networks. CompBio also identified categories related to signaling pathways, such as Wnt, Hedgehog, BMP, TGFβ, or Notch, suggesting that CELF1 may regulate these key actors of lens development and disease [1].

**Figure 3.**
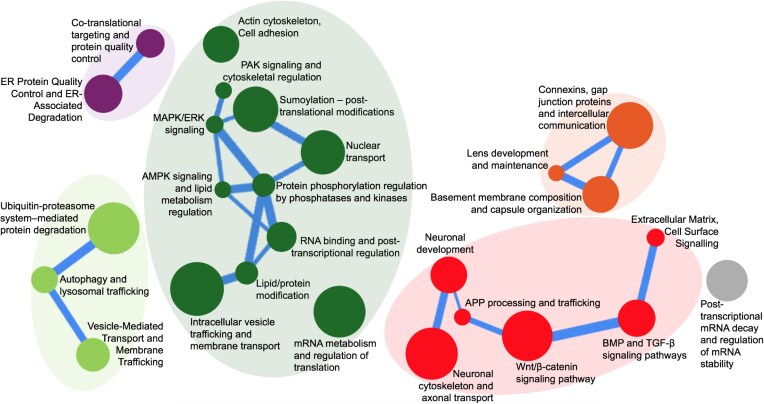
Enriched biological themes identified by CompBio in CELF1 iCLIP-seq datasets in the lens. CompBio-based analysis of 636 genes (found in CLIP1 and/or CLIP2) identifies enriched biological themes. CompBio does *de novo* analysis of the test list of candidates using the published scientific literature covering >30 million PubMed abstracts and 3 million full-text articles. Circles denote nodes that represent enriched themes while the edges between nodes indicate that genes are shared to denote the different themes they connect. The size of a circle is based on the number of genes that contribute to the node and the thickness of an edge denotes the amount of overlapping genes. In the different clusters, the themes that are related are represented with similar colors.

Among the most unexpected findings was the enrichment of neuronal categories, including “neuronal differentiation” and “neuronal cytoskeleton, axonal transport, axonal guidance.” Notably, several transcripts contributing to these themes (e.g. *Kif1a, Pafah1b1, Reep1, Rtn3, Tubb2a*, and *Vamp2*) are overexpressed in *Celf1*-cKO lenses [38], suggesting that CELF1 contributes to maintaining lens identity by suppressing inappropriate expression of neuronal genes. This interpretation is consistent with previous studies that propose active mechanisms that prevent neuronal gene expression in the lens [54, 55]. Overall, CompBio analysis shows that CELF1 directly regulates transcripts involved in multiple aspects of lens biology, including differentiation, cell architecture, signaling, and maintenance of tissue identity.

### CELF1 ligands encode key regulators of lens development

To investigate the functional relevance for lens development and diseases of CELF1 ligands, we first assessed their lens specificity using the iSyTE 2.0 database. The lens-enrichment module of iSyTE 2.0 compares gene expression in the lens and in the whole body, to identify lens-specific and lens-enriched genes [56]. Figures 4A-A' show higher lens-enrichment for CELF1 ligands than for non-ligands. Hence, CELF1 ligands are enriched in genes that show a preferential expression in the lens, suggesting their functional importance in lens biology.

**Figure 4.**
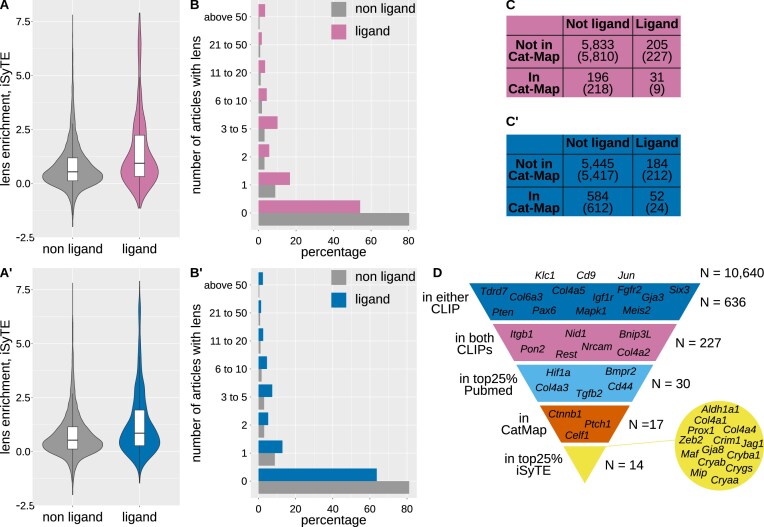
CELF1 ligands are enriched for genes involved in lens development and disease. (**A**-**A′**) Lens expression enrichment of CELF1 ligands versus non-ligands, using iSyTE 2.0. (A) intersection (*P* = 1.6e-09, Wilcoxon test), (A′) union (*P* = 1.6e-16). (**B**-**B′**) Number of PubMed citations per gene retrieved with “[gene name] AND lens” for CELF1 ligands and non-ligands. (B) intersection (*P* = 2.7e-21, chi-square test comparing the distribution of ligands and non-ligands between no citation and at least one citation), (B′) union (*P* = 9.5e-25). (**C**-**C′**) Contingency tables comparing CELF1 ligands and non-ligands for inclusion in Cat-Map. (C) intersection (*P* = 6.5e-15, chi-square test), (C′) union (*P* = 1.4e-09, chi-square test). (**D**) Gene prioritization workflow (left) and the number of gene passing each selection step (right), with example genes filtered out at each stage.

As an orthogonal approach, we performed PubMed queries combining each gene with the keyword “lens.” We found that ligand genes were more frequently co-mentioned with “lens” than non-ligands, including in multiple publications, both considering the intersection or the union (Fig. 4B-B′). Hence, CELF1 ligands are enriched in genes that have already been investigated for their roles in lens biology.

We next examined their association with cataract. According to Cat-Map [10, 11], 538 genes are implicated in cataract (February 2025 release). Of these 31 (intersection) and 52 (union) overlap with CELF1 ligands, significantly more than expected by chance (Fig. 4C-C′). This implicates CELF1 ligands in lens disease.

### A multistep pipeline prioritizes key CELF1 ligands for functional studies

To prioritize ligands for functional validation, we applied a multistep filtering funnel integrating binding, disease association, and lens-specific expression data (Fig. 4D). As previously described, starting from 10 640 genes expressed in the lens, we first retained 636 transcripts identified as CELF1 ligands in at least one iCLIP-seq experiment. Restricting the analysis to ligands detected in both datasets yielded 227 high-confidence targets. Of them, we retained 30 genes that are in the upper quarter of the number of publications in a PubMed search with “lens.” Filtering for inclusion in Cat-Map next reduced the list to 17 genes. These include *Celf1* itself, *Ptch1* which encodes a Sonic Hedgehog receptor involved in radiation-induced cataract in animal models [57, 58], or *Ctnnb1*, which encodes a major component of the Wnt signaling pathway with established roles in lens development and diseases [1]. Finally, the last filtration relying on a high lens-enrichment of expression (top quarter) retained 14 genes (Fig. 4D). They encode diverse classes of proteins, including crystallins, extracellular matrix components, transmembrane protein involved in intercellular communication and transport (JAG1, CRIM1, MIP, GJA8/connexin-50) or transcription factors (MAF, PROX1, ZEB2). This refined gene set provided a focused framework for subsequent functional analyses aimed at identifying key mediators of the *Celf1* lens phenotype.

### *Maf* and *Gja8* expression levels are elevated in mouse lenses in the absence of CELF1

Among the 14 high-priority gene ligands of CELF1, we previously demonstrated that *Prox1* is downregulated by CELF1 in mouse lenses. It is also the case of *Pax6*, found in CLIP1 [37]. For further analysis, we focused on two additional genes with distinct molecular functions: *Gja8* and *Maf*, which encode a connexin and a transcription factor, respectively. In control postnatal day 15 (P15) lenses, GJA8 localizes along the membranes of lens fiber cells, which are organized in a regular pattern along the antero-posterior axis (Fig. 5A, upper panels). In contrast, in P15 *Celf1*-cKO lenses, while GJA8 remains membrane-associated, the cellular architecture is disrupted. This disorganization is consistent with previously reported vacuolization and defective cell-to-cell junctions in *Celf1*-deficient lenses [34]. Moreover, GJA8 accumulates in distinct punctate structures rather than displaying its typical uniform distribution along the cell membranes. Notably, GJA8 signal intensity is markedly increased compared to controls, indicating that *Gja8* expression is higher in *Celf1*-cKO lenses (Fig. 5A).

**Figure 5.**
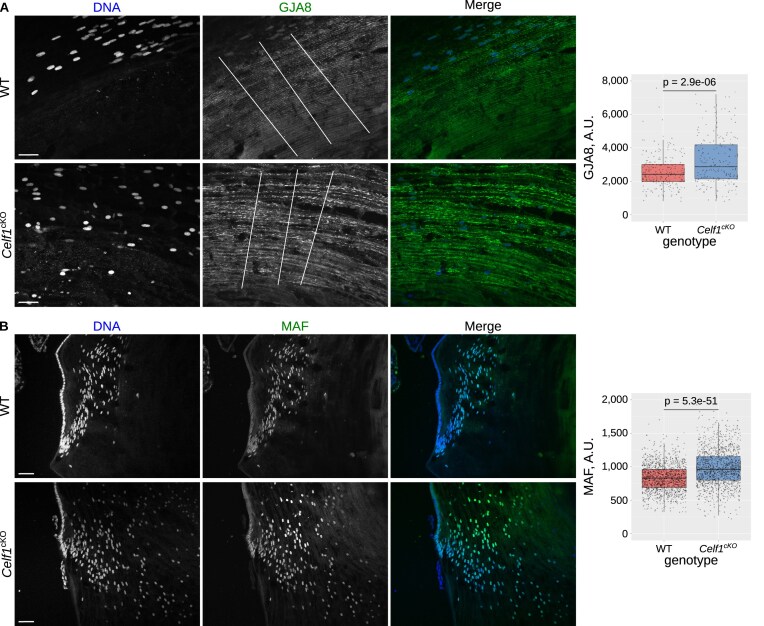
Increased expression of *Gja8* and *Maf* in *Celf1*-cKO mouse lenses. (**A**) Immunofluorescence of GJA8 in control (upper panel) and *Celf1*-cKO (lower panel) P15 mouse lenses. Right panel, quantification of fluorescence intensity at local maxima along the lines drawn in the second-left panel. Each dot represents one local maximum. (**B**) Immunofluorescence of MAF in control (upper panel) and *Celf1*-cKO (lower panel) P15 mouse lenses. Right panel, quantification of MAF intensity in 300–500 DAPI-defined nuclei from several sections. *P*, Wilcoxon tests. Each dot represents one nucleus. Scale bars: 50 µm (A) and 75 µm (B).

At the same developmental stage, MAF is absent from lens epithelial cells but begins to accumulate in the nuclei of cells entering the transition zone (Fig. 5B). As cells move deeper into the lens and differentiate into fiber cells, MAF protein levels decline in maturing fiber cells. However, in *Celf1*-cKO lenses, MAF persists longer within internalized nuclei and displays stronger nuclear staining compared to controls, reflecting elevated MAF protein levels in the absence of CELF1 (Fig. 5B).

### CELF1 represses *Maf* expression in 21EM15 cells

To further validate the regulation of *Maf* by CELF1, we conducted a rescue experiment using the 21EM15 mouse lens cell line, which includes a stable *Celf1* knock-down derivative [34]. As previously described [37], CELF1 depletion leads to increased PAX6 and PROX1 protein levels (Supplementary Fig. S4). While GJA8 was undetectable in these cells where the gene is not expressed, immunostaining confirmed elevated MAF protein levels in *Celf1*-depleted cells, paralleling the observations made *in vivo* (Fig. 6A). To determine whether reintroduction of CELF1 could reverse this effect, we transiently transfected sh*Celf1* 21EM15 with a vector encoding a CELF1-Cherry fusion protein. Transfected cells could be readily distinguished from non-transfected cells by Cherry labeling (arrows in Fig. 6B). We then compared MAF levels between non-transfected sh*Celf1* cells and Cherry-positive cells in transfected cells, where the CELF1-Cherry fusion protein is present. Nuclear MAF protein levels were significantly reduced in the presence of CELF1 (Fig. 6B). Together, these data show that loss of CELF1 increases MAF levels, whereas re-expression of *Celf1* restores lower MAF protein levels. Together with iCLIP and reporter assay data, these findings demonstrate that CELF1 directly represses *Maf* expression.

**Figure 6.**
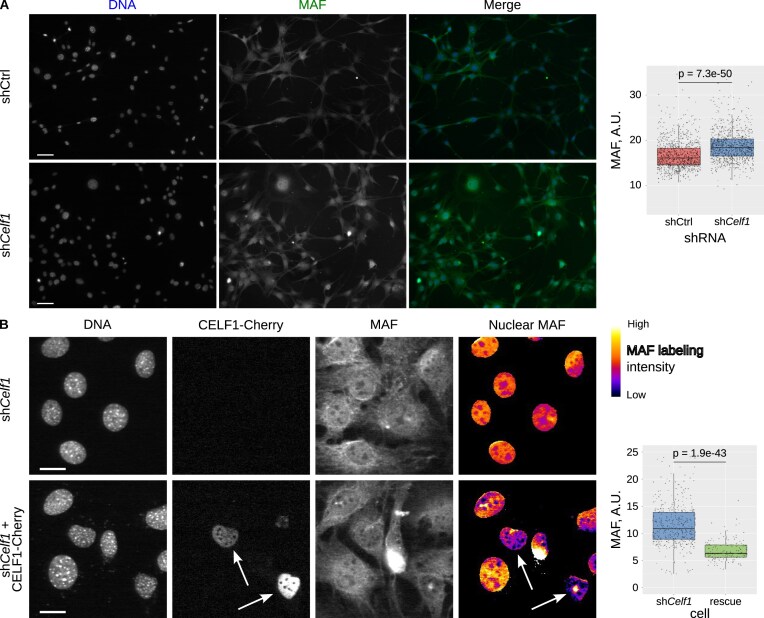
CELF1 represses *Maf* expression in 21EM15 cells. (**A**) Immunofluorescence staining for MAF in control (upper panels) and sh*Celf1* knockdown (lower panels) 21EM15 cells. (**B**) Immunofluorescence staining for MAF in non-transfected sh*Celf1* knockdown 21EM15 cells (upper panels) and in sh*Celf1* knockdown 21EM15 cells transfected with a CELF1-Cherry expression vector (lower panels). Cells that express CELF1-Cherry are indicated by arrows. “Nuclear MAF” shows MAF fluorescence intensity in a false-color scale. Right panels quantifications of MAF fluorescence intensity in 300–500 DAPI-defined nuclei from multiple fields. Each dot represents one nucleus. *P*, Wilcoxon tests. Scale bars: 50 µm (A) and 10 µm (B).

### Lens defects in *Xenopus* larvae overexpressing *maf*

Our data demonstrate that CELF1 directly binds to the *Maf* 3′UTR (Fig. 1), that this 3′UTR represses translation in a CELF1-dependent manner (Fig. 2), and that *Maf* is overexpressed in both cultured cells and lenses lacking CELF1 (Figs 5 and 6). To evaluate whether Maf overexpression contributes to the cataract phenotype observed in Celf1-cKO mice [34], we turned to *X. laevis*, a well-established model for lens development and disease [59].

We injected *X. laevis* embryos at the 4-cell stage in one dorsal blastomere with *X. laevis* maf mRNA together with a lineage tracer. After two days of development, embryos were sorted based on tracer fluorescence to identify the injected side. Larvae were analyzed at 3 days post-fertilization. Figure 7A–C illustrates representative phenotypes of stage 39 larvae. Overall body morphology appears normal following maf overexpression. The non-injected eyes are comparable to those of uninjected controls and tracer-only injected embryos (Fig. 7D). In contrast, eyes on the injected side display various external defects, including microphtalmia with a greyish lens (Fig. 7A) a ventral retinal gap suggestive of coloboma (Fig. 7B), or a small, recessed eye (Fig. 7C). Quantitative analysis of multiple larvae revealed that approximately 70% of *maf*-injected larvae exhibited externally visible eye abnormalities, compared to a low percentage in tracer-only controls (Fig. 7E).

**Figure 7.**
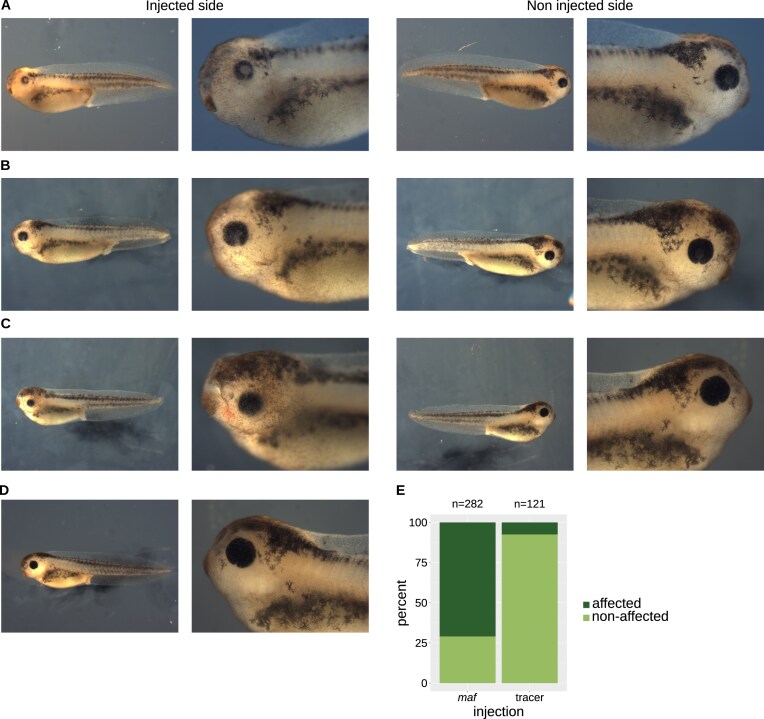
Eye abnormalities induced by *maf* overexpression in *X. laevis* larvae. (**A–C**) Representative external views of stage 39 *X. larvae* injected at the four-cell stage into one dorsal blastomere with *Maf* mRNA and a fluorescent lineage tracer. Left panels, injected side (identified by fluorescence); right panels, uninjected control side. (**D**) External view of a sibling embryo injected with lineage tracer only (injected side). (**E**) Quantification of externally visible eye defects. *P* = 5.1e-31, chi-square test.

To further characterize the defects induced by *maf* overexpression, we performed histological analysis of larvae injected with lineage tracer alone or with *maf* mRNA (Fig. 8A–D). In tracer only larvae, eye morphology and lens structure are similar on both sides, confirming that the lineage tracer does not interfere with lens development (Fig. 8A). In contrast, while the non-injected sides of *maf*-injected embryos resemble those of controls (Fig. 8B–D), the injected sides display notable abnormalities (Fig. 8B′–D′). In some cases, retinal layering is preserved (Fig. 8B′), whereas in others it is severely disorganized (Fig. 8C′ and D′). Lens defects are consistently more severe, in that the injected sides exhibit smaller lenses (Fig. 8B′), impaired fiber cell differentiation as evidenced by reduced eosin staining (Fig. 8C′), or nearly complete lens absence, with only rudimentary remains (Fig. 8D′). To quantify these defects, we measured eye and lens areas on serial sections by calculating the product of the minor and major diameters. Both are significantly reduced on the injected side (Fig. 8E and F); however, lens growth is more severely impaired than overall eye growth, as reflected by a significantly lower lens-to-eye area ratio (Fig. 8G).

**Figure 8.**
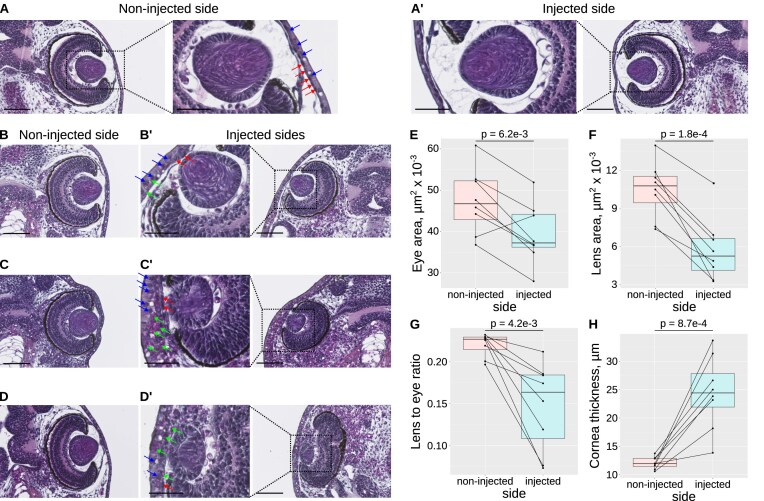
*maf* overexpression disrupts lens and corneal structure in *X. laevis* larvae A-A', Histological sections of a stage 39 *Xenopus* larva injected at the four-cell stage into one dorsal blastomere with the lineage tracer alone. (**A**) Non-injected control side. (**A′**) Injected side. (**B–D**) Representative sections of sibling larvae co-injected with *maf* mRNA and the lineage tracer. In (**B′–D′**), higher magnification, arrows indicate: blue, nuclei of squamous corneal epithelial cells; red, nuclei of corneal presumptive endothelial cells; green, infiltrated cells. Scale bars: 100 µm (low magnification), 50 µm (high magnification). (**E–H**) quantifications of eye area (E), lens area (F), lens-to-eye area ratio (G), and corneal thickness (H), on injected versus non-injected sides (*N* = 8 larvae). *P*, paired Student’s *t*-test.

In addition to reduced eye and lens size, maf overexpression disrupts the organization of the presumptive cornea, the tissues separating the lens from the external environment. In tracer-only controls, the cornea displays a clearly defined outer squamous epithelium (blue arrows) and an inner presumptive endothelium (red arrows, Fig. 8A), consistent with previous reports [60]. Figures 8C and 8B′–D′ show that this organization is lost in maf-injected larvae, which exhibit a prismatic corneal epithelium, a fragmented or discontinuous endothelium, and dense eosin-labeled cells suggesting infiltration of blood cells coming from peripheral tissue (green arrows). We also observed consistent thickening of the presumptive cornea (Fig. 8H), likely contributing to the externally visible “sunken” eye phenotype (e.g. Fig. 7C). Previous studies using surgical or toxin-induced lens ablation have shown that lens-derived signals are essential for corneal differentiation [61–63]. Several pathways, including Semaphorin and TGF-β signaling, have been implicated [64, 65]. Notably, lens-specific overexpression of TGF-β1 induces corneal thickening [65]. Therefore, the corneal defects seen in maf-overexpressing *Xenopus* larvae likely result from disrupted lens development. Collectively, these findings demonstrate that maf overexpression disrupts lens development in *Xenopus*, supporting the hypothesis that Maf derepression in Celf1-deficient mouse lenses contributes to cataract formation.

## Discussion

In this study, we generated the first transcriptome-wide map of CELF1–RNA interactions in an intact vertebrate lens. By combining iCLIP-seq with transcriptomic analyses, reporter assays, and *in vivo* functional studies, we show that CELF1 acts as a major post-transcriptional repressor during lens development. CELF1 binding is enriched within the 3′UTRs of transcripts encoding key regulators of lens differentiation and cataract-associated genes, and its targets are preferentially upregulated in *Celf1*-deficient lenses. Functional analyses further demonstrate that CELF1 represses gene expression through both mRNA destabilization and translational inhibition. Among the identified targets, *Maf* emerged as a particularly compelling candidate: its expression is increased following CELF1 depletion in mouse lenses and lens cells, and its overexpression in *X. laevis* is sufficient to disrupt lens morphogenesis. Together, these findings support a model in which CELF1 safeguards lens development by preventing inappropriate accumulation of developmental regulators and other lens-relevant transcripts.

In human, several genetic studies have established a strong association between mutations in the *MAF* gene and congenital cataracts [66–76]. These mutations are typically point mutations located within the MAF DNA-binding domain and are often inherited in an autosomal dominant manner. In addition to cataracts, affected individuals frequently exhibit broader ocular developmental abnormalities, including Peters anomaly, Aymé–Gripp Syndrome, coloboma, amblyopa, or microcornea. In mouse models, two independent *Maf* mutations causing congenital cataracts were also found to be dominant or semi-dominant [77, 78]. This supports the notion that cataract-associated *MAF* mutations are predominantly gain-of-function. This is exemplified by the D90V mutation in mice, which results in increased promoter activity of target genes [77].

Beyond these naturally occuring mutations, targeted gene inactivation strategies have been employed in mice to elucidate the role of *Maf* in lens development. Germline *Maf* knockout mice exhibit microphtalmia, with severe defects in lens morphogenesis resulting from impaired fiber cell elongation, and reduced expression of crystallin genes [79, 80]. This underscores MAF's function as a transcriptional regulator of crystallin genes, a conclusion further supported by luciferase reporter assays demonstrating that wild-type MAF, but not disease-associated mutants, activates promoters of four crystallin genes *CRYAA, CRYBA4, CRYBA1*, and *CRYGA* [69]. Conditional inactivation of *Maf* in adult mice using a tamoxifen-inducible Cre system also leads to cataract formation, although with reduced severity compared to germline deletion [81]. This indicates that MAF is critical not only during lens development but also for maintaining lens structures in adults. Hence, both gain-of-function and loss-of-function (targeted disruption) mutations of *MAF* cause lens diseases in mammals, including cataracts.

Here, using *Xenopus* as a model system, we show that overexpression of wild-type *maf* via mRNA injection is sufficient to induce lens defects. While gene overexpression may appear mechanistically similar to gain-of-function mutations, it can also have inhibitory effects by disturbing dosage-sensitive gene networks or competing for cofactors [82]. Thus, our finding that overexpression of wild-type *maf* leads to lens malformations unveils a distinct pathogenic mechanism involving *MAF* in cataract, complementary to traditional loss-of-function or point mutation models.

This raises an important question: to what extent can gene overexpression or ectopic expression be generalized as a pathogenic mechanism beyond Maf? We propose that the systematic identification of CELF1-bound RNAs in the lens, as reported in this study, provides a valuable set of candidates to address this question. We demonstrate for the first time, at a transcriptome-wide level and in a specific organ, that CELF1 functions as a global post-transcriptional repressor, reducing both mRNA abundance and translation efficiency. As a result, CELF1 deficiency leads to widespread upregulation of its mRNA ligands, including *Maf*. It is reasonable to hypothesize that overexpression of at least a subset of these ligands, especially those with known roles in lens development, may contribute with *Maf* to cataract formation in Celf1 conditional knockout mice. In mouse models, ectopic expression of *Pax6* or *Foxe3* (both driven by the *Cryaa* promoter) in lens fiber cells results in lens defects [83, 84], and the latter has also been analyzed by microarrays to identify the impact of its overexpression on the transcriptome [83]. A similar model for *Maf* would inform on the impact of its overexpression on the lens. Further, such a model would also provide new insights into the mRNAs misexpressed in *Celf1*-cKO lens, by indicating the mRNA targets that can be attributed to CELF1-based direct control, and the mRNA targets that likely result from indirect control by CELF1, via overexpression of *Maf*. It is noted that CELF1 deficiency causes elevation of PAX6, PROX1 [37] and MAF (this study) in the lens, and it will be important to examine the specific contributions of the higher dosage of these key transcription factors to the lens defects. It should also be noted that while *MAF* mutations have been linked to cataract in animal models and/or humans since >25 years, there are still significant knowledge-gaps regarding this key transcription factor, both in terms of its upstream and downstream regulation, which our findings help to address. Future studies that systematically test the impact of overexpressing individual CELF1 targets in model systems like *Xenopus* or lens organoids [59, 85] could uncover novel genes whose dosage must be tightly regulated to maintain lens integrity. Such efforts would expand our understanding of lens biology and cataract pathogenesis, moving beyond conventional gene disruption studies and providing a new framework for functional validation of cataract candidate genes.

## Supplementary Material

ugag037_Supplemental_Files

## Data Availability

The iCLIP-seq data have been deposited in NCBI’s Gene Expression Omnibus and are accessible as GSE313211. The R script generated to treat the data has been deposited in Zenodo with unlimited access, https://doi.org/10.5281/zenodo.20830191.

## Notes

Present address: Institut de recherche interdisciplinaire en Biologie humaine et moléculaire (IRIBHM), Faculté de Médecine, Campus Erasme, 1070 Bruxelles, Belgium
