# Supplementary material for: Mapping of CELF1–RNA interactions reveals post-transcriptional control of lens development
Source: NAR Mol Med. 2026 Aug 5;3(3):ugag037. doi: 10.1093/narmme/ugag037 (PMC13454838; doi:10.1093/narmme/ugag037)
Supplement: Supplementary file 1 [file ugag037_supplemental_files.zip › supp_data_forcompil.pdf]

FIGURE S1

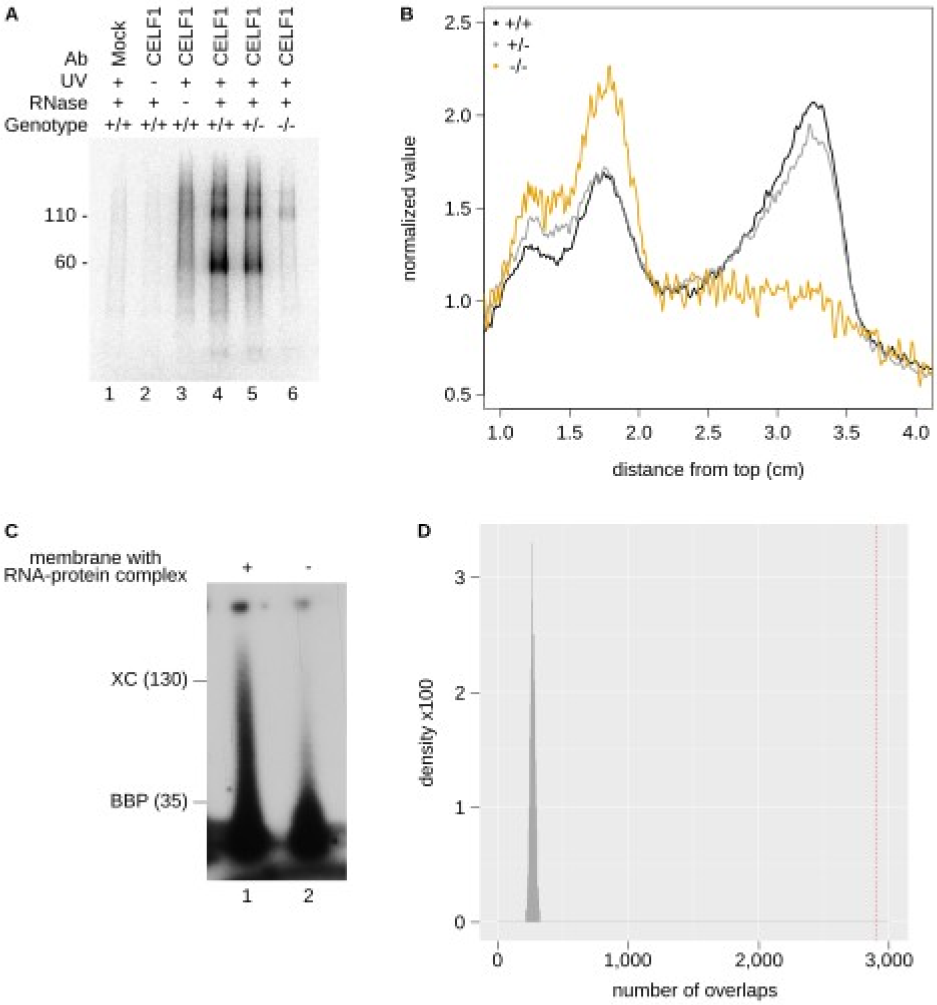

Figure S1. Data for iCLIP-seq

A, Freshly dissected mouse wild-type (lanes 1-4), *Celf1*$^{+/-}$ (lane 5) or *Celf1*$^{-/-}$ (lane 6) mouse lenses were UV-irradiated (lanes 1, 3-6), lyzed, and submitted to an immunoprecipitation with a mock (lane 1) or an anti-CELF1 antibody (lanes 2-6). RNA were submitted to gentle RNase T1 treatment (lanes 1-2, 4-6) and radiolabelled by ligation of a radioactive DNA primer. Protein-RNA complexes were resolved by neutral electrophoresis, transfered to pure nitrocellulose membranes, and autoradiographed. RNA-protein complexes around 60 kDa were only detected starting from mouse lenses that express *Celf1*, with anti-CELF1 antibody, following UV and RNase treatments (lanes 4-5). **B**, Quantification of Figure S1A, lanes 4-6. The ~60 kDa radioactive signal (migration distance 3.0 to 3.5 cm) is absent starting from *Celf1*$^{-/-}$ lenses. **C**, RNA were eluted by proteinase treatment from the part of the nitrocellulose membrane around 60 kDa shown in Figure S1A, lane 4, and further labeled with [γ-^{32}P] ATP (lane 1). Lane 2 is the same treatment but with no nitrocellulose membrane added. Molecular weights are infered from the migration of Xylene Cyanol (130 nucleotides) and Bromophenol Blue (35 nucleotides). 100-150 nucleotide long RNAs are only detected in lane 1. **D**, Number of CLIP2 clusters that are included (at least one overlapping nucleotide) within CLIP1 clusters. The red dotted line indicates what is observed (N = 2,902). The grey peak represents the density of CLIP2 clusters overlapping with CLIP1 clusters after randomly shuffling them 1,000 times within the same genes (average value = 268). While the exact p-value cannot be calculated with this approach, finding that none of the number of overlaps following 1,000 independent random replacements exceeds the observed value implies that $p < 10^{-3}$.

FIGURE S2

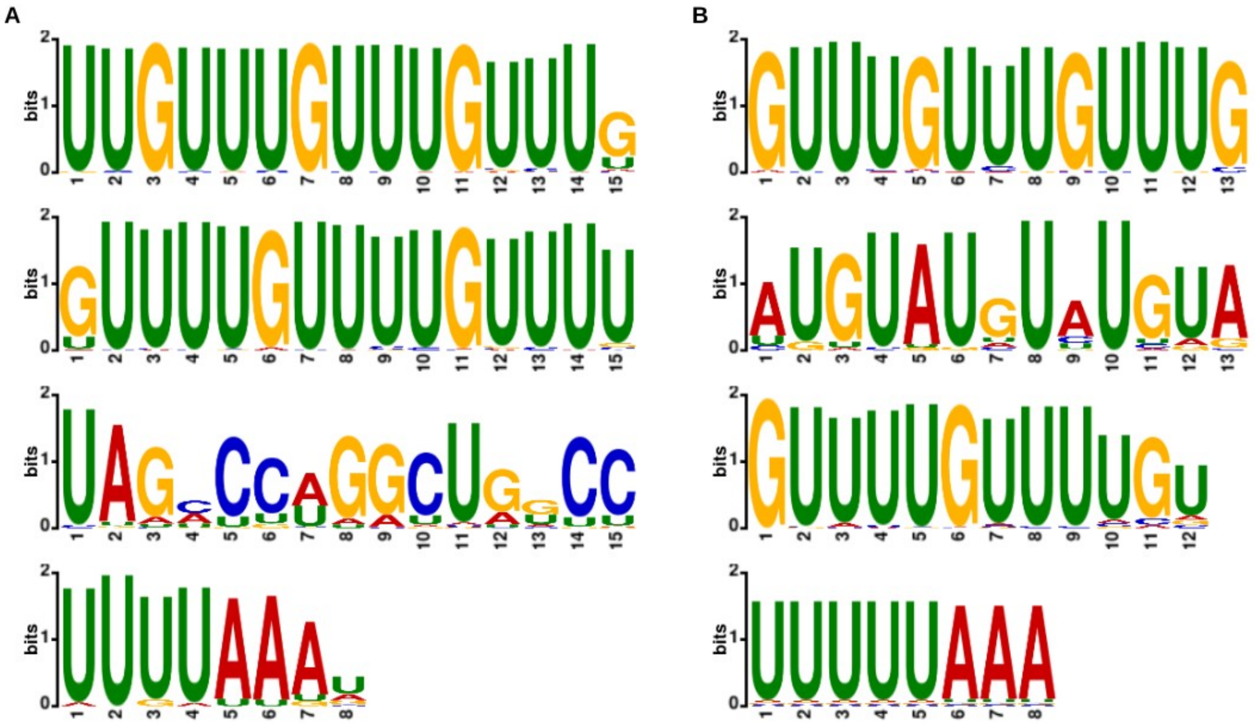

Figure S2. Most enriched motifs in CLIP clusters.
A, CLIP1. **B**, CLIP2. Motifs are ranked from top to bottom by increasing p-value. The most enriched motif is a (GUUU) repeat in both CLIP1 and CLIP2. Variations around this theme include the presence of 4 Us instead of 3 (2nd and 3rd most enriched motifs in CLIP1 and CLIP2, respectively), or the replacement of the second U by an A (2nd most enriched motif in CLIP2).

FIGURE S3

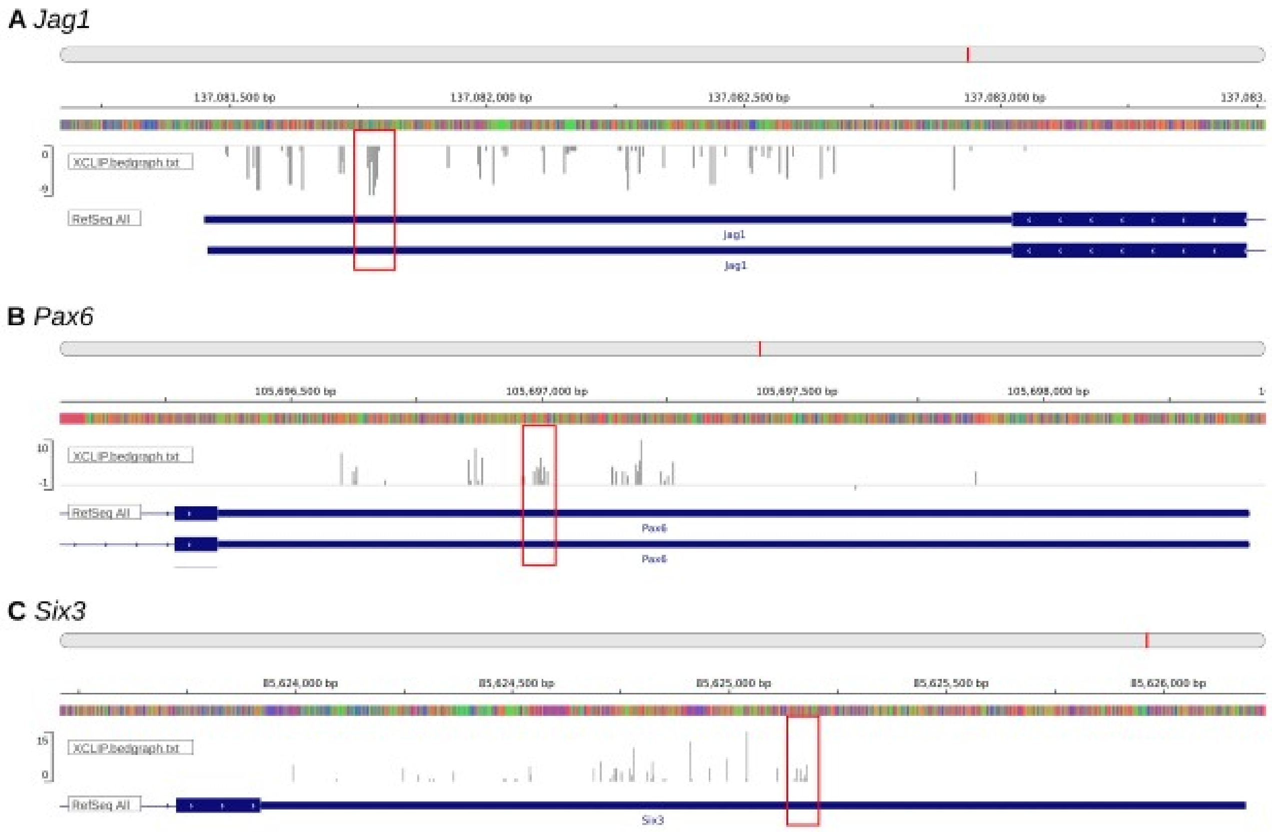

Figure S3. CELF1 binding clusters in *Jag1*, *Pax6*, *Six3*

CELF1 binding clusters identified in the 3'UTRs of *Jag1* (**A**), *Pax6* (**B**) and *Six3* (**C**). Red rectangles indicated high-density regions of CELF1-binding clusters that were deleted in the mutant constructs used in Figure 2F-G.

FIGURE S4

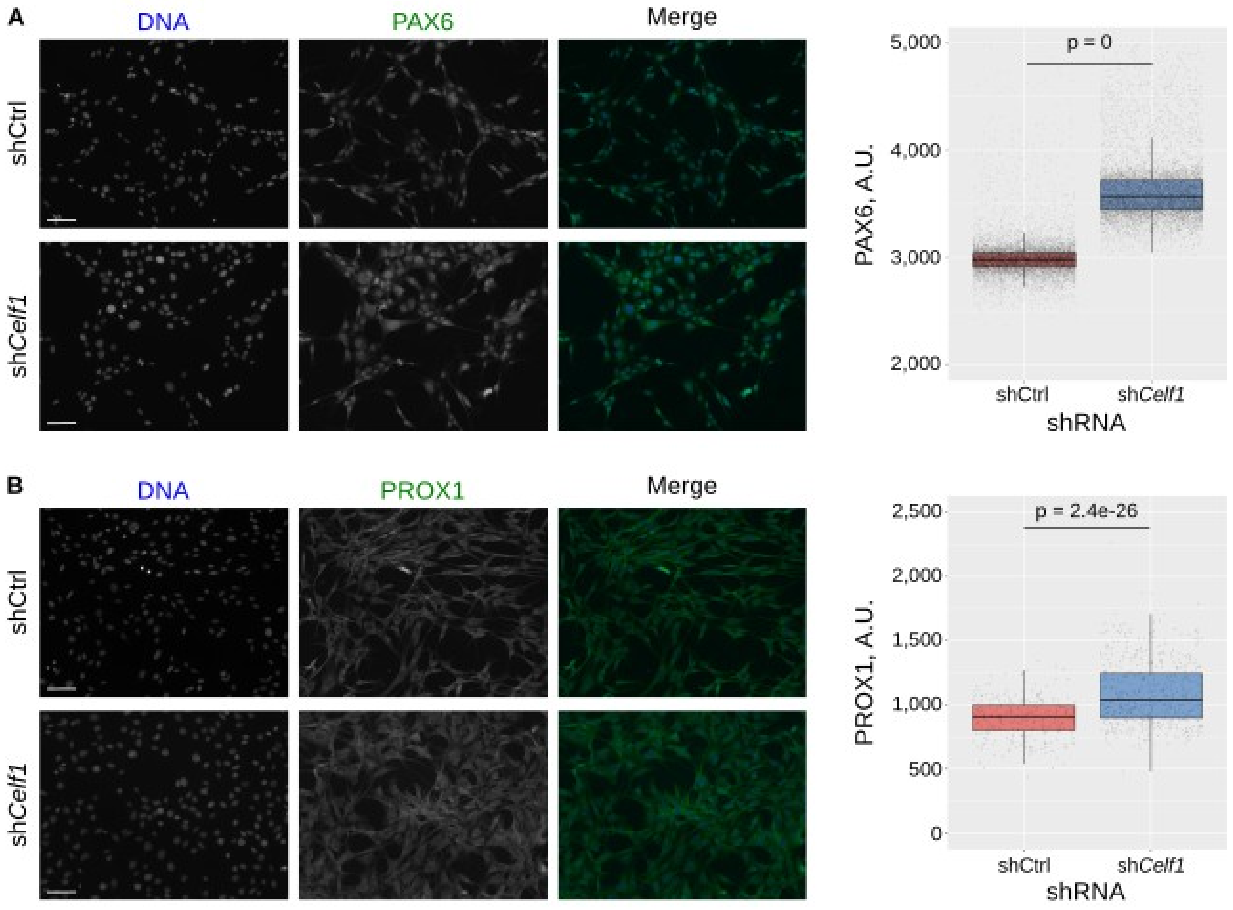

Figure S4. Expression of *PAx6* and *Prox1* in mouse lenses and cultured lens cells
Immunofluorescence of PAX6 (**A**) and PROX1 (**B**) in control (upper panel) and sh*Celf1* knockdown (lower panel) 21EM15 cells. Right panel, quantification of PAX6 and PROX1 intensities in 300-500 DAPI-defined nuclei across multiple fields. Scale bars 50 µm.

LEGENDS TO SUPPLEMENTARY TABLES

Table S1. iCLIP-seq data

Total score, sum of all cluster scores within the 3'UTR. maxUGU, number of UGU within the 3'UTR cluster containing the highest number of UGU. Exp, expression (CPM). Pubmed, number of citations combining gene name with the keyword "lens". HeLa, gene previously identified as a CELF1 ligand in HeLa cells [44]. catmap, gene present in the Cat-Map database, February 2025 release [10,11]. isyte, maximal enrichment score in iSyTE 2.0 [54].

Table S2. Primer sequences

Sequences of the primers used for constructs, with their usage.
